# Toxicity of Manganese Titanate on Rat Vital Organ Mitochondria

**DOI:** 10.22037/ijpr.2019.1100639

**Published:** 2019

**Authors:** Maliheh Entezari, Fatemeh Ghanbary

**Affiliations:** a *Department of Genetics, Faculty of Advanced Science and Technology, Tehran Medical Sciences, Islamic Azad University, Tehran, Iran.*; b *Department of Chemistry, Faculty of Basic Sciences, Mahabad Branch, Islamic Azad University, Mahabad, Iran.*; 1 M. E. and F. G. contributed equally to this work.

**Keywords:** Manganese titanate, Oxidative phosphorylation, Isolation of mitochondria, Mitochondria toxicity, Cytochrome c release

## Abstract

The TiO2, which is a main material in the field of photocatalytic reactions, includes rutile and anatase phase. Titanium dioxide has possessed notice due to its promising applications in the environmental photocatalytic degradation of pollutants of organic compound in waste water and utilization of solar energy. The nanosized manganese titanate (pyrophanite) MnTiO3 was collected by oxidation of Mn(OH)2 with TiO2 powder in cetyltrimethylammonium bromide (CTAB) micelle solutions and the calcinations of the produced powders. Therefore, it was decided to determine the Mechanistic mitochondria toxicity of nanoparticles towards liver, kidney, heart, and brain via new and reliable methods. Our results showed that nanoparticles induced mitochondria dysfunction via an increase in ROS production and membrane potential collapse, correlated to cytochrome c release. Also, increased disturbance in oxidative phosphorylation was also shown by the decrease in ATP. Recent studies have suggested that nanoparticles leading to cytosolic release of lysosomal content, and ultimately apoptosis. This study suggests that mitochondrial oxidative stress and impairment of oxidative phosphorylation in vital organ Mitochondria may play a key role in manganese titanate toxicity.

## Introduction

Nanomaterials are extremely small in size and possess a large surface area per unit of volume (1, 2). Nanomaterials have specific physicochemical characteristics and are created intentionally for different application. The different commercial uses of nanoparticles for novel applications are increasing exponentially. Many nanomaterials synthesized as commercial products are introduced during our daily lives. Researcher used Nanoparticles instead nanomaterials, with commercial and industrial applications, such as personal skin and hair care products, sunscreens, pigments, coatings, ceramic products, and paints (2). The range of nanotechnology products is wide and they can be classified into several various compound categories, including metals, carbon, silica, metal oxides, and semiconductor nanomaterials (3). 

The toxicity of nanomaterials has been studied in various biological systems, both in cell line systems and different organisms, such as humans, rodents, and aquatic species such as zebrafish, catfish, algae and macrophages (4-5). Manganese titanate (MnTiO3) has recently attracted much attention for its strong absorption in the visible region which may be propitious to the utilization of solar energy and photocatalysis (4-7). Also they are important due to their weak magnetism and semi conductivity (8). Zeolites are crystalline aluminosilicates containing pores and channels of molecular dimensions that are widely used in industry as ion exchange resins, molecular sieves, sorbents, and catalysts (9). 

Appropriate particles for such usage need to be biocompatible with the ability to control size, morphology, and rate of biodegradation, as well as drug loading and release (1). In this pilot study, we perform a MnTiO3 on vital tissue. Mitochondria were isolated from the mouse for the first time. Vital organs such as liver, kidney, heart, and brain are selected in other organs. To elucidate the possible mechanisms of cytotoxicity, a mitochondrial Reactive oxygen species (ROS) formation, mitochondria membrane potential, ATP assay, and Cytochrome c expulsion assay, were quantitatively assessed for exposure to nanoparticle and compared with control groups.

So, we assumed that mitochondrial dysfunction may be contributed in manganese titanate on rat vital organ Mitochondria. Regarding that the exact mechanism by which manganese titanate on rat vital organ Mitochondria may produce toxicity is unclear, so this study aimed to investigate the effect of manganese titanate on oxidative phosphorylation, mitochondrial ROS production, and also cytochrome c release using isolated rat vital organ mitochondria to more elucidating the mechanism of the manganese titanate toxicity in an attempt to find out mechanistic ways to prevent toxicity of manganese titanate.

## Experimental

*Materials*

All chemicals and reagents were purchased from Sigma-Aldrich (Taufkrichen, Germany) at best commercial grade available. We used for ROS and mitochondrial membrane potential (MMP) of Sigma Chemical Co. (St. Louis, MO, USA) and we used Quantikines Rat/Mouse Cytochrome c Immunoassay Kit (Minneapolis, MN) for cytochrome c release.

*Instrument*

Incubator 37 ºC, Sensor CO2 Sanyo IR, japan MCO 17A1; vapor bath Stark eliwellewpc 800T, UKA; refrigerated centrifugation, model of Sanyo, Harrier 18/80, Japan; Spectrophotometric UV/Visible, Shimadzu 160 ABB, Japan; Floremetry, Shimadzu RF-5000, Japan; digital scale Japan, Shimadzu 20 E8 330H, Japan; Shaker, REAX2000, Iran; ELISA reader (In finite 200 M, TECAN). 

*Animals*

Male Wistar rats (250–300 g) were fed with a normal standard chow diet and tap waterad libitum. we used 6 number of animals each of groups in our study. All experiments were conducted according to the ethical standards and protocols approved by the Committee of Animal Experimentation of Shahid Beheshti University of Medical Sciences, Tehran, Iran. We type the study *in-vivo* plain. After the animals were decapitated, the liver, kidney, heart, and brain were quickly excised, pooled, and rinsed using isotonic saline buffer. These samples were used for the isolation of mitochondria as described below.

*Experimental design*

The animals were classified into two groups as follows: group 1: control group the normal saline injection, group 2: treatment group (give nanoparticle) and 0.003 g nanoparticle are weighted and suspended in normal saline. finally, once IP injection.

*Preparation of mitochondria*

The mitochondria isolation used Mitochondria Isolation Kit Catalog Number MITOISO1. This kit enables the fast and easy isolation of an enriched mitochondrial fraction from animal tissues such as liver, kidney, heart, and brain.

**Table 1:** Effect of nanoparticle induced ROS formation in isolated liver, kidney, heart and brainmitochondria (0.5 mg/mL). ROS formation was determined by RF-5000U fluorescence spectrophotometer using 2′,7′-dichlorofluorescein diacetate (DCFH-DA) as described in materials and methods and demonstrated as fluorescence intensity of DCF. Values represented as mean ± SEM (n = 6). *P < 0.05; **P < 0.01 and ***P < 0.001 compared with control mitochondria

**Groups**	**ROS formation**			
	**Control**		**Test**	
	**15 (min)**	**60 (min)**	**15 (min)**	**60 (min)**
Brain	905.3 ± 0.91	907.2 ± 0.49	1001.3 ± 1.8**	1001.6 ± 2.3**
Heart	1003 ± 32	1010 ± 22	1004.3 ± 33*	1009 ± 34*
Liver	1016.6 ± 35	1016.6 ± 36	1023.6 ± 32	4584 ± 29***
kidney	1245 ± 38	1274.6 ± 35	1288 ± 38	1306 ± 25*

**Table 2:** Effect of nanoparticle on mitochondrial membrane potential MMP collapse (ΔΨ%) in isolated, isolated liver, kidney, heart and brain mitochondria (0.5 mg/mL). membrane potential collapse (ΔΨ%) was measured by RF-5000U fluorescence spectrophotometer using Rhodamine 123 as described in Experimental. The values are expressed as means ± SEM (n = 6). *P < 0.05; **P < 0.01 and ***P

**Groups**	**Mitochondrial membrane potential**			
	**Control**		**Test**	
	**15 (min)**	**60 (min)**	**15 (min)**	**60 (min)**
Brain	1048 ± 2.4	1054.3 ± 2	1089 ± 1.6*	2003 ± 3.5*
Heart	1016.3 ± 34	1025.3 ± 34	2514 ± 97**	2205.6 ± 70**
Liver	1324 ± 24	1351.3 ± 57	2415 ± 52**	2710.3 ± 13**
Kidney	1612.3 ± 48	1623 ± 49	2926 ± 12**	3861.3 ± 11**

< 0.001 compared with control mitochondria.

**Figure 1:**
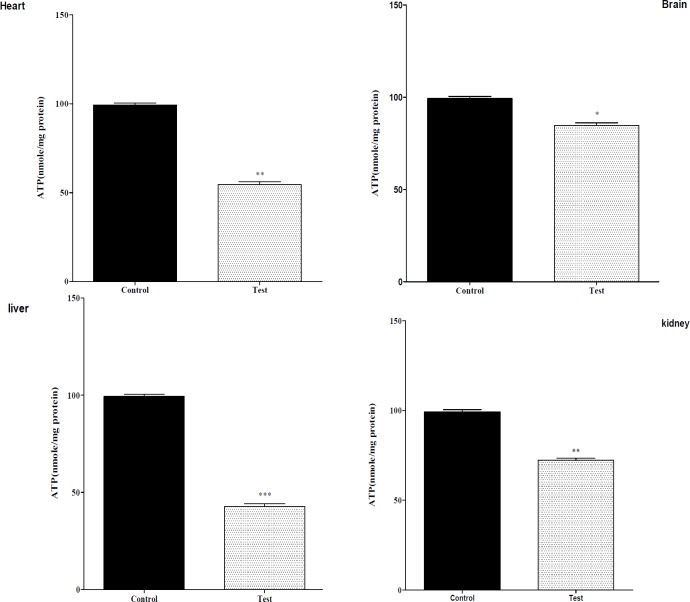
Effect of nanoparticle on mitochondrial ATP level on isolated liver, kidney, heart and brain mitochondria (0.5 mg/mL). Isolated mitochondria (0.5 mg/mL) were incubated with silk fibroin and ATP level were determined using Luciferin/Luciferase Enzyme System as described in Experimental. Values represented as mean ± SEM (n = 6). *P < 0.05; **P < 0.01 and ***P < 0.001 compared with control mitochondria

**Figure 2:**
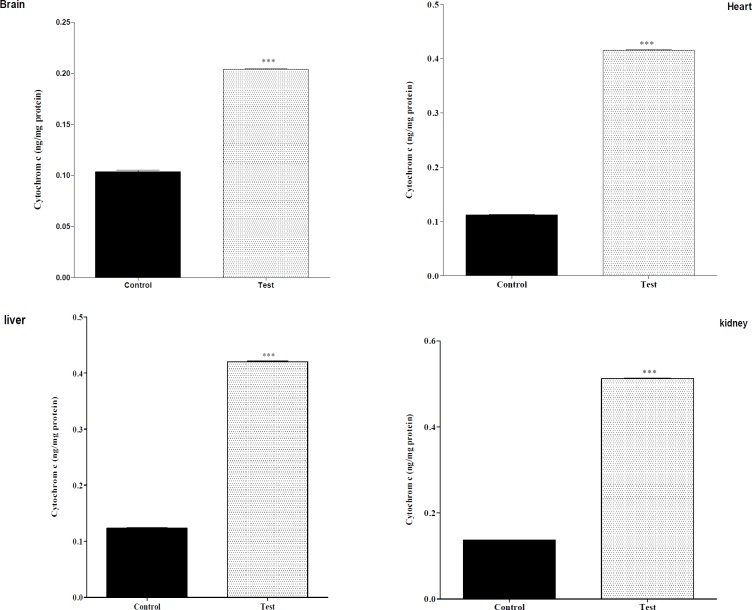
Effect of nanoparticle on cytochrome c release mitochondrial on liver, kidney, heart and brain mitochondria (0.5 mg/mL). the mitochondria were incubated for 1 h with various concentrations of silk fibroin. The amount of released cytochrome c from mitochondria was determined after 1 h of incubation using Cytochrome c ELISA kit as described in Materials. Values represented as mean ± SEM (n = 6). ^*^P < 0.05; ^**^P < 0.01 and ^***^P < 0.001 compared with control mitochondria

*Protein concentration*

Protein concentration of Mitochondrial was determined by the Coomassie blue protein-binding method using bovine serum albumin (BSA) as the standard (10).

*Quantification of mitochondrial ROS level*

The Fluorometric Intracellular ROS Assay Kit provides a sensitive, one-step fluorometric assay to detect intracellular ROS (especially superoxide and hydroxyl radicals) in live cells with 1 h incubation. ROS react with a fluorogenic sensor was localized to the cytoplasm, resulting in a fluorometric product (ex = 490/em = 520 nm) proportional to the amount of ROS present.

*Mitochondrial membrane potential assay*

The Fluorometric Mitochondrial membrane potential Assay Kit provides a sensitive, one-step fluorometric assay to detect Mitochondrial membrane potential in live cells with a 1 h incubation. The mitochondrial uptake of the cationic fluorescent dye, rhodamine 123, was used for determination of MMP by fluorometric (ex = 490/em = 525 nm). we used Rhodamine 123 (rh123) Instead jc.

*Assay of ATP level*

The ATP levels were measured using Luciferin/Luciferase Enzyme system (Tafreshi *et al.* 2007). Bioluminescence intensity was measured using Sirius tube luminometer (Berthold Detection System, Germany).

*The amount of cytochrome c released to the medium*

From isolated mitochondria was determined at 450 nm according to the instructions provided by the manufacturer of the Quantikines Rat/Mouse Cytochrome c Immunoassay Kit (Minneapolis, MN). All analysis stages were carried out using an ELISA reader (InfiniteM 200, TECAN) at desired concentrations in all groups.

*Statistical analysis*

The results are presented as means SEM. All statistical analyses were performed using the Prism version 6 software in our study. Also the assays were performed in triplicate and means used for statistical analysis. The normality test was Kolmogorov-Smirnov (K-S) test and statistical significance was determined using the one-way ANOVA test, followed by the post-hoc Tukey test. Statistical significance was set at *P* < 0.05.

## Results

*The effect of in-vivo mn-tio3 treatment on mitochondrial ROS production*

Effects of nanoparticleon mitochondrial ROS production as shown in Table 1, nanoparticle induced significant ROS formation in rat liver, kidney, heart, and brain mitochondria. As demonstrated, there was a significant increase expression of dichlorofluorescein (DCF). Our study noted that nanoparticle-induced mitochondrial ROS production in once injection.

*The effect of in-vivo mn-tio3 treatment on mitochondrial MMP*

As shown in Table 2, nanoparticle significantly decreased MMP in all mitochondrial test groups obtained from liver, kidney, heart, and brain. there was a significant increase expression of rhodamin123.

*The effect of in-vivo mn-tio3 treatment on mitochondrial ATP level*

Mitochondrial electron transfer chain is required for ATP production, since nanoparticle exposure impairs the mitochondrial electron transfer chain; it was therefore decided to measure ATP levels in isolated mitochondria following the injection nanoparticle. As shown in Figure 1, all nanoparticle significantly decreased mitochondrial ATP levels in isolated liver, kidney, heart mitochondria. 

*The effect of in-vivo mn-tio3 treatment on mitochondrial cytochrome c release*

As shown in Figure 2, there was a significant difference in the release of cytochrome c between control mitochondria and test groups [liver, kidney, heart and brain], whereas this was observed for the all of mitochondria with nanoparticle and cytochrome c release.

## Discussion

Nanoscience has matured significantly during the last decade as it has transitioned from bench top science to applied technology. Presently, nanomaterials are used in a wide variety of commercial products such as electronic components, sports equipment, sun creams and biomedical applications. The substantial differences in physicochemical properties of nanomaterials compared to the bulk phase has been recognized in numerous scientific and technological areas (2). Nanomedicine is a new field of science based on the significantly enhanced properties of nanoparticles (NPs) that make possible the early diagnosis and new treatments for catastrophic diseases, such as multiple sclerosis, atherosclerosis, and cancer (11). In the previous study, also concluded that mitochondria play a key role in the apoptosis of cells by nanoparticles via decline in the Bcl-2/Bax ratio accompanied with the loss of MMP and excess cytosolic calcium (12). In this investigation several important findings warrant additional discussion. First, based on our results the cell viability extremely decrease level in brain, liver, heart and kidney. a significant decrease in function of complex II activity following injection of nanoparticles in animal rats and isolation cells and mitochondria from vital organs Such as brain, liver, heart and kidney induced, stress oxidative (13). Cytotoxicity from ROS can be more pronounced in the central nervous system (CNS) due to the high content of unsaturated fatty acids, which are susceptible to peroxidation (14). ROS also play a role in the development of vasculopathies, including those that define atherosclerosis, hypertension, and restenosis after angioplasty and liver or kidney failure (15). This suggests that the order of sensitivity of these tissues against nanoparticle toxicity is different level. Second, increase in ROS formation was observed in all investigated tissues following after nanoparticle injection in animal and isolated mitochondria confirming the probable involvement of mitochondrial ROS in nanoparticle-induced toxicity mechanisms. ROS generated by nanoparticles and probably playing important role in the initiation of membranelipid peroxidation (LPO). LPO is a marker of oxidative stress (16). on the reference studies seems that oxidation of mitochondrial lipid membranes might result in disruption of mitochondrial electron transfer chain and consequently collapse of MMP and cytochrome c release (16). results confirmed collapse of MMP events lead to increased ROS formation and disturbance in mitochondrial electron transfer. Others study showed that nanoparticles induced ROS production not only leads to lipid peroxidation but also GSH oxidation, both events damage mitochondrial membrane integrity and mitochondrial permeability transition (MPT) pore opening leading to cell death signaling (17). Therefore, GSH as cellular antioxidant protects cells against peroxidation of lipid membrane and thus, GSH gets depleted in this reaction. In this study, the level of ATP was diminished in treated groups. Our data also showed that impairment of electron transfer chain results in reduced ability for ATP synthesis. 

The highest fall in ATP levels were found in all of the isolated tissue mitochondria. In addition, depletion in ATP levels promotes a switch from apoptotic to necrotic cell death; however, ATP depletion to greater than 80% leads to necrosis (18). We showed that mitochondria were injured after nanoparticle injection animal and so isolation of cells due to down regulation of an ATP synthase subunit. This led to cell dysfunction. In addition, nanoparticles at induced apoptosis and necrosis occurs (18). Mitochondrial depolarization or MPT pores opening would be expected to produce ATP depletion due to conversion of mitochondrial ATPsyntase to ATPase, followed by cytochrome c release in cytosol. In this study we found release of cytochrome c in all of the groups.
